# Short- and long-term predictions of chaotic flows and extreme events: a physics-constrained reservoir computing approach

**DOI:** 10.1098/rspa.2021.0135

**Published:** 2021-09

**Authors:** N. A. K. Doan, W. Polifke, L. Magri

**Affiliations:** ^1^ Institute for Advanced Study, Technical University of Munich, Lichtenbergstrasse 2a, 85748 Garching, Germany; ^2^ Department of Mechanical Engineering, Technical University of Munich, Boltzmannstrasse 15, 85748 Garching, Germany; ^3^ Department of Aeronautics, Imperial College London, South Kensington, London, UK; ^4^ The Alan Turing Institute, 96 Euston Road, London NW1 2DB, UK; ^5^ Faculty of Aerospace Engineering, Delft University of Technology, Kluyverweg 1, 2629 HS Delft, The Netherlands

**Keywords:** reservoir computing, machine learning, extreme events, chaotic flows

## Abstract

We propose a physics-constrained machine learning method—based on reservoir computing—to time-accurately predict extreme events and long-term velocity statistics in a model of chaotic flow. The method leverages the strengths of two different approaches: empirical modelling based on reservoir computing, which *learns* the chaotic dynamics from data only, and physical modelling based on conservation laws. This enables the reservoir computing framework to output physical predictions when training data are unavailable. We show that the combination of the two approaches is able to accurately reproduce the velocity statistics, and to predict the occurrence and amplitude of extreme events in a model of self-sustaining process in turbulence. In this flow, the extreme events are abrupt transitions from turbulent to quasi-laminar states, which are deterministic phenomena that cannot be traditionally predicted because of chaos. Furthermore, the physics-constrained machine learning method is shown to be robust with respect to noise. This work opens up new possibilities for synergistically enhancing data-driven methods with physical knowledge for the time-accurate prediction of chaotic flows.

## 1. Introduction

Many fluid dynamics systems exhibit extreme events, which are violent and sudden changes of a flow from the average evolution [1]. Examples of extreme events in fluids are oceanic rogue waves [2], extreme patterns in atmospheric and climate science [3,4], intermittency in turbulence [5] and thermoacoustic instabilities in aeroengines and rocket motors [6], to name only a few. Although governed by deterministic equations such as conservation laws, extreme events occur in a seemingly random way. The time-accurate detection and prediction of the dynamics of extreme events can be achieved for a short time scale, which is the predictability time [7]. This is a roadblock for the *time-accurate* prediction because, after the predictability time, a tiny difference between the initial conditions, such as floating-point errors, is exponentially amplified. This is popularly known as the butterfly effect in chaos theory [8]. Because of this, the time-accurate prediction of extreme events remains an open problem [1].

Most state-of-the-art predictive approaches rely on statistical methods. Extreme value theory [9] and large deviation theory [10] characterize the probability of an event and the heavy tail of the probability density function, which can be used to compute the initial conditions with the highest probability of transitioning towards extreme values. Statistical approaches successfully identified precursors of turbulent channel flow relaminarization [11] and of nonlinear rogue waves [12]. Another approach for the prediction of extreme events is data-driven. Machine learning has proved successful at predicting the dynamics of some chaotic flows with accurate short-term predictions and long-term statistics. Vlachas *et al*. [13] used a long short-term memory (LSTM) network, which is a type of recurrent neural network (RNN) [14], to predict the evolution of the Kuramoto–Sivashinsky equation and of a barotropic climate model. They showed that the proposed neural network had a good short-term accuracy, and converged towards the correct invariant measures. A similar architecture was employed in [15] to simulate the evolution of a model of shear turbulence showing that it was capable of reproducing the moments of the velocity statistics. Another type of RNN—the echo state network (ESN) [16,17]—based on reservoir computing, can successfully learn the chaotic dynamics beyond the predictability time for short-term predictions [18–20] and ergodic averages [21]. Data-driven methods have the capability of predicting chaotic dynamics, but it is still an open question whether they can be robustly used to predict extreme events. Wan *et al*. [22] tackled this problem with a hybrid approach that combined a reduced-order model with an LSTM. They predicted the evolution of dissipation events in the Kolmogorov flow and intermittent transitions between two flow regimes in a model of barotropic flow. Sapsis [23] combined large deviation theory with a data-driven method to efficiently characterize the heavy tail of the distribution in the Kolmogorov flow.

Statistical methods provide a robust framework to identify precursors and calculate the probability of extreme events, but they do not provide a robust way to time-accurately predict their occurrence and amplitude. On the other hand, recurrent neural networks, such as LSTMs and ESNs, can predict the dynamics of chaotic systems by learning temporal patterns in data only, but, because they are fully data driven, they provide solutions that may violate the governing physical laws, such as momentum conservation. In fluid mechanics, machine-learned solutions, however, should obey physical principles such as momentum and mass conservation. This calls for embedding physical and domain knowledge in data-driven methods [20,24–27].

To achieve this physical embedding, various methods have been proposed in the past [24]. On the one hand, several works have explored the possibility of directly using physical systems as computational basis for deep learning [28–30]. Specifically, in these works, the dynamics of the Karman vortex sheet, or of nonlinear waves, were leveraged as a computational reservoir, or RNN, which were then used to compute and predict the dynamics of other systems. This demonstrated the strong computational capability of such physical machine learning approaches. This approach worked particularly well when the physical system used as a reservoir had similarities with the system to be studied, for example, in flows [30].

On the other hand, another approach consists of including physical knowledge within traditional machine learning architectures [20,24,25,31,32], or modifying the target that the machine learning architecture has to learn. This automatically ensures physical conservation, such as by learning the Lagrangian of the system [33]. In [25,32], a physics-informed neural network was developed to approximate the solution of PDEs [25], or infer unmeasured variables from measurable quantities in flows [32]. This approach relied on a feedforward neural network combined with a physics-based loss to estimate the quantities to infer that are not present in the training data. This enhancement of the loss function with physical knowledge was also explored in [31], where the optimization process to identify the dynamics of a reduced order flow model was constrained with energy-preserving constraints in the Sparse Identification of Nonlinear Dynamics (SINDy) framework. The models obtained with those energy-preserving constraints compared favourably with reduced order models obtained with traditional Galerkin projection methods.

Further combinations of machine learning tools with physics-based methods were explored in the combination of computational fluid dynamics with machine learning [34,35]. For example, in [34], neural networks were used to discover improved spatial discretizations of PDEs on a coarse grid, which allowed for the accuracy to be equivalent to the accuracy of a finer grid, thereby enabling the accurate resolution of PDEs on a coarse mesh. Following similar ideas, in [35], convolutional neural networks were used within a direct numerical simulation solver to learn the correction required for a coarse simulation to reach the accuracy of a finer simulation allowing for a speed-up of the calculation while improving the accuracy drastically.

The discussion above highlights that additional research is necessary to enable purely data-driven machine learning approaches to time-accurately predict the evolution of chaotic systems during extreme events, therefore requiring the integration of some physical knowledge. In this paper, we propose a specific method based on a physics-based modification of the loss function used during the training of the machine learning model.

Specifically, the objective of this paper is to propose a machine learning method that (i) produces physical solutions to time-accurately predict extreme events in a qualitative model of shear turbulence and (ii) reproduces the long-term statistics. To achieve this objective, we will start from a reservoir computing framework based on the ESN and show that, by embedding physical knowledge during the training, the proposed *physics-informed* machine learning framework can achieve short- and long-term time-accurate predictions. This paper studies for the first time the application of such a hybrid physics-informed reservoir computing framework to the extreme event predictions where the inclusion of such physics-based knowledge can produce a marked improvement compared to purely data-driven approaches given that extreme events are generally rare within any dataset. The flow configuration is presented in §2. The physics-informed echo state network (PI-ESN) is developed in §3. Results are shown in §4. The long-term statistical behaviour of the network is discussed in §4a. Short-term predictions of extreme events are analysed in §4b. The robustness of the overall architecture with respect to noise is presented in §4c. A final discussion with future directions concludes the paper.

## 2. Extreme events in a model of chaotic flow

We regard the chaotic flow as an autonomous dynamical system

2.1 $$\dot{y}=N(y),\;y(0)=y_{0}$$

where $\dot{\left(\right)}$ is the temporal derivative; and N is a deterministic nonlinear differential operator, which encapsulates the numerical discretization of the spatial derivatives and boundary conditions (if any).

The flow is governed by momentum and mass conservation laws, i.e. the Navier–Stokes equations, which were reduced in form by Moehlis, Faisst and Eckhart (MFE) [36]. This model, which was inspired by earlier works [37,38], provides the nonlinear operator N. This is called the MFE model for brevity. The MFE model captures the qualitative features of the transition from turbulence to quasi-laminar states such as the exponential distribution of turbulent lifetimes. The velocity field in the model is decomposed as

2.2 $$v(x,t)=\sum_{i=1}^{9}a_{i}(t)v_{i}(x),$$

where ***v_i_***(***x***) are spatial Fourier modes (or combinations of them) [36]. The Navier–Stokes equations are projected onto ***v_i_***(***x***) to yield nine ordinary differential equations for the modes’ amplitudes, *a_i_*, which are nonlinearly coupled. Consequently, the state vector is $y={\left\{a_{i}\right\}}_{1}^{9}$. All the variables are non-dimensional [36]. Physically, $v_{1}$ is the laminar profile mode; ***v***_2_ is the streak mode; ***v***_3_ is the downstream vortex mode; ***v***_4_ and ***v***_5_ are the spanwise flow modes; ***v***_6_ and ***v***_7_ are the normal vortex modes; ***v***_8_ is the three-dimensional mode; and ***v***_9_ is the modification of the mean profile caused by turbulence. The flow has a fixed point *a*_1_ = 1, *a*_2_ = · · · = *a*_9_ = 0, which is a laminar state. The electronic supplementary material reports the expressions for ***v_i_*** and the equations for *a*_*i*_ [36]. Detailed analysis of the MFE model was performed by Kim & Moehlis [39] and Joglekar *et al.* [40].

**Figure 1.**
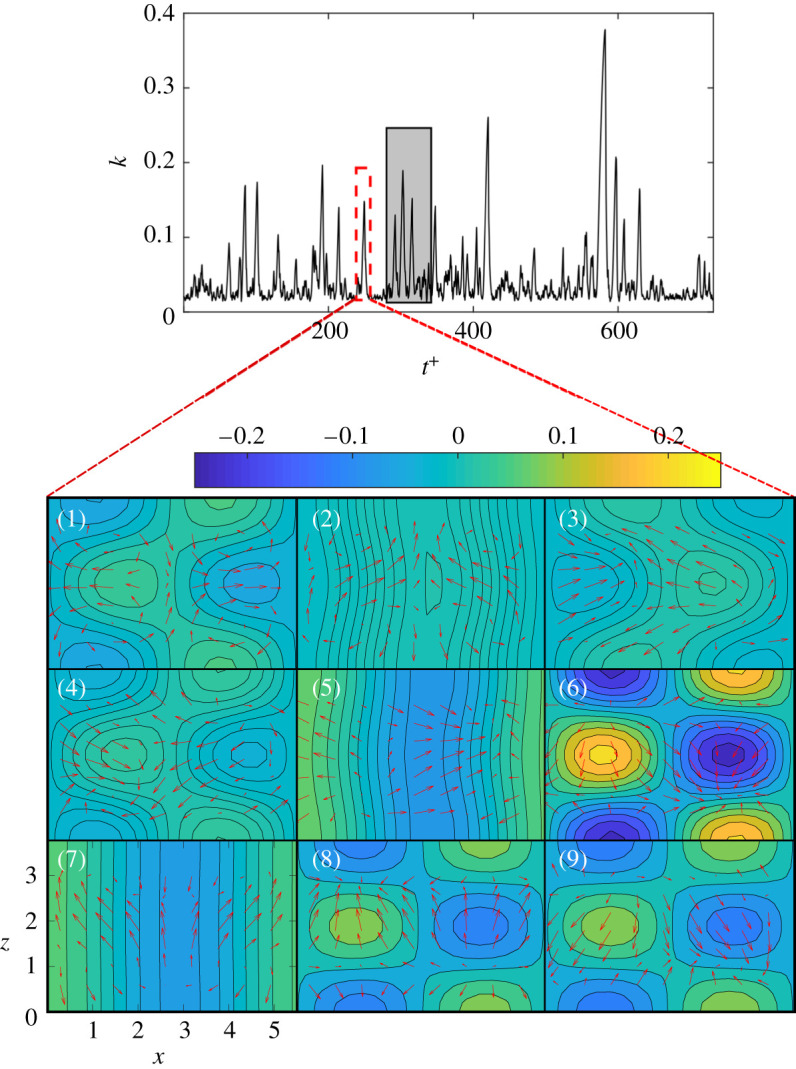
Top panel: kinetic energy, *k*. The grey box indicates the training window of the PI-ESN. Bottom panel: velocity field in the mid-*y* plane. The arrows indicate the in-plane velocity (*x*–*z* directions), and the colour maps indicate the out-of-plane velocity. *t*^+^ is the time normalized by the largest Lyapunov exponent. (Online version in colour.)

The flow under investigation is incompressible. The domain is a cuboid of size *L*_*x*_ × *L*_*y*_ × *L*_*z*_ between two infinite parallel walls at *y* = 0 and *y* = *L*_*y*_, which are periodic in the *x*- and *z*-directions. The domain size is *L*_*x*_ = 1.75*π*, *L*_*y*_ = 2 and *L*_*z*_ = 1.2*π*. The Reynolds number is 600. A sinusoidal volume force is applied in the *y*-direction. The initial condition (electronic supplementary material) is such that the turbulent flow displays chaotic bursts between the fully turbulent and quasi-laminar states. These are the extreme turbulent events we wish to predict. The governing equations are integrated in time with a 4th-order Runge–Kutta scheme with a time step Δ*t* = 0.25 to provide the evolution of the nine modes, *a*_*i*_, from *t* = 0 to *t* = 80 000. The evolution of the kinetic energy, $k=0.5\sum_{i=1}^{9}a_{i}^{2}$, is shown in figure 1 for the first 30 000 timesteps. The time is normalized by the largest Lyapunov exponent, *λ*_max_ ≈ 0.0244, which is calculated as the average logarithmic error growth rate between two nearby trajectories [7]. The Lyapunov time scale, $\lambda_{\max}^{-1}$, provides an estimate of the predictability time, which is used to define the non-dimensional time

2.3 $$t^{+}=\frac{t}{\lambda_{\max}^{-1}}.$$

The kinetic energy, *k*, has sudden large bursts that arise from a chaotic oscillation with a small amplitude. Each burst is a quasi-relaminarization event, which occurs in three phases (figure 1): (i) the originally laminar velocity profile becomes unstable and breaks down into vortices due to the shear imposed by the volume force (panels 5–7); (ii) the vortices align to become streaks (panels 8–9 and 1–2); and (iii) the streaks break down leading to flow relaminarization (panels 3–5).

## 3. Physics-constrained reservoir computing

**Figure 2.**
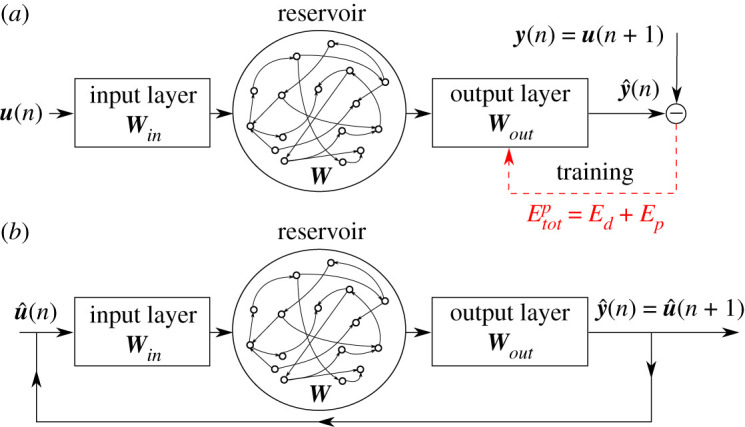
PI-ESN during (*a*) training and (*b*) prediction. (Online version in colour.)

To learn the reduced-order dynamics of shear turbulence, we constrain the physical knowledge of the governing equations into a reservoir computing data-driven method based on the ESN [16,17]: the PI-ESN [20]. A schematic of the network is shown in figure 2.

We have training data with an input time series $u(n)\in R^{N_{u}}$ and a target time series $y(n)\in R^{N_{y}}$, where *n* = 0, 1, 2, …, *N*_*t*_ are the discrete time instants that span from 0 to *T* = *N*_*t*_Δ*t*. During prediction, the target at time *n* becomes the input at time *n* + 1, i.e. ***u***(*n* + 1) = ***y***(*n*). The training of the PI-ESN is achieved by (i) minimizing the error between the prediction, $\hat{y}(n)$, and the target data ***y***(*n*) when the PI-ESN is excited with the input, ***u***(*n*) (figure 2*a*), and (ii) enforcing that the prediction does not violate the physical constraints. To enforce (ii), we observe that a solution of the chaotic flow, $y={\left\{a_{i}\right\}}_{1}^{9}$, is such that the *physical error* (also known as the residual) is zero, i.e. $F(y)\equiv \dot{y}-N(y)=0$. To estimate the physical error beyond the training data, after exciting the PI-ESN with the training data, the PI-ESN is then looped back to its input (figure 2*b*) to obtain the predictions ${\left\{\hat{y}(N_{t}+p)\right\}}_{p=1}^{N_{p}}$ in the time window with no training data, (*T* + Δ*t*) ≤ *t* ≤ (*T* + *N*_*p*_Δ*t*). The number of collocation points, *N*_*p*_, is user-defined. The physical error $F(\hat{y}(N_{t}+p))$ is evaluated to train the PI-ESN such that the sum of (i) the physical error between the prediction and the available data from *t* = 0 to *t* = *T*, *E*_*d*_, and (ii) the physical error for *t* > *T*, *E*_*p*_, is minimized. Mathematically, we wish to find $\hat{y}(n)$ for *n* = 0, 1, …, *N*_*t*_ + *N*_*p*_ that minimizes

3.1 $$E_{\text{tot}}^{P}=\underset{E_{d}}{\underbrace{\frac{1}{N_{t}+1}\sum_{n=0}^{N_{t}}||\hat{y}(n)-y(n)|{|}^{2}}}+\underset{E_{p}}{\underbrace{\frac{1}{N_{p}}\sum_{p=1}^{N_{p}}||F(\hat{y}(N_{t}+p))|{|}^{2}}},$$

where ∣∣⋅∣∣ is the Euclidean norm. The PI-ESN is straightforward to implement because it requires only cheap residual calculations at the collocation points, i.e. it does not require the exact solution. Practically, the estimation of the time-derivative in $F(\hat{y})$ is performed with finite difference and, for a specific time-step *k*, the physical error is estimated as $F(\hat{y})\approx ({\hat{y}}_{k+1}-{\hat{y}}_{k})/\Delta t-N({\hat{y}}_{k})$. Therefore, this loss function penalizes solutions that do not fulfil the governing equations (to a numerical tolerance), similarly to physics-informed feedforward neural networks to approximate the solution of PDEs [25] or infer unmeasured quantities [32]. Here, we apply this physics-based loss to an echo state network to obtain a time-accurate surrogate model capable of autonomously reproducing the dynamics of the system.

### (a) Network architecture

The architecture of the PI-ESN follows that of the ESN, which consists of an input matrix $W_{\text{in}}\in R^{N_{x}\times N_{u}}$, which is a sparse matrix; a *reservoir* that contains *N*_*x*_ neurons that are connected by the recurrent weight matrix $W\in R^{N_{x}\times N_{x}}$, which is another sparse matrix; and the output matrix $W_{\text{out}}\in R^{N_{y}\times N_{x}}$. The input time series, ***u***(*n*), is connected to the reservoir through ***W***_in_ to excite the states of the neurons, ***x***, as

3.2 $$x(n+1)=\tanh(Wx(n)+W_{\text{in}}u(n+1)),$$

where tanh( · ) is the activation function. The output of the PI-ESN, $\hat{y}(n)$, is computed by linear combination of the reservoir states as $\hat{y}(n)=W_{\text{out}}x(n)$. The matrices ***W***_in_ and ***W*** are randomly generated and fixed [41]. Only ***W***_out_ is trained to minimize (3.1). Following [19], each row of ***W***_in_ has only one non-zero element, which is drawn from a uniform distribution over [−*σ*_in_, *σ*_in_]; ***W*** has an average connectivity 〈*d*〉, whose non-zero elements are drawn from a uniform distribution over the interval [−1, 1]; and ***W*** is scaled such that its largest eigenvalue is Λ≤1, which ensures the echo state property [41]. The same quadratic transformation of the reservoir state before readout as in [19] is also applied.

The training of the PI-ESN is achieved in two steps. First, the network is initialized by an output matrix, ***W***_out_, that minimizes a data-only cost functional $E_{\text{tot}}^{NP}=E_{d}+\gamma ||w_{\text{out},i}|{|}^{2}$, where *γ* is a Tikhonov regularization factor and ***w***_out,*i*_ denotes the *i*th row of ***W***_out_. This is the output matrix of the conventional ESN [18]. Second, the physical error (3.1) is minimized with the L-BFGS method [42], which is a quasi-Newton optimization algorithm. On the one hand, the proposed PI-ESN architecture has similarities to an Elman-RNN architecture because the PI-ESN can be interpreted as a three-layer RNN trained with a gradient-based optimizer. On the other hand, the PI-ESN is different from an Elmann-RNN because it employs a physics-based loss function, whose additional training is limited to ***W***_out_ to reduce the computational cost. In principle, other parts of the ESN, such as the input and recurrent weight matrices, ***W***_in_ and ***W***, can be trained. This is not attempted here because the training of ***W***_out_ is sufficient to show a substantial improvement in the prediction accuracy (§4).

## 4. Results

A grid search provides the hyperparameters Λ=0.9, *σ*_in_ = 1.0, 〈*d*〉 = 3, *γ* = 10^−6^, which enable accurate predictions in the range of *N*_*x*_ = [500, 3000] neurons (electronic supplementary material). These optimal hyperparameters are determined for a data-only ESN, and are re-used for the PI-ESN, unless mentioned otherwise, to provide a base assessment of the improvement that can be obtained by introducing the physics-based loss. Only *t* = 2500 time units (equivalent to *t*^+^ ≈ 61) in the window *t* = [11 500, 14 000] (equivalent to *t*^+^ ≈ [280, 341] in the grey box of figure 1) are used for training. The data beyond this time window are used for validation only. We use *N*_*p*_ = 5000 collocation points (equivalent to *t* = 1250 or *t*^+^ ≈ 30.5), which provide a sufficient number of predictions beyond the training data with a relatively low computational time.

### (a) Long-term statistical behaviour

**Figure 3.**
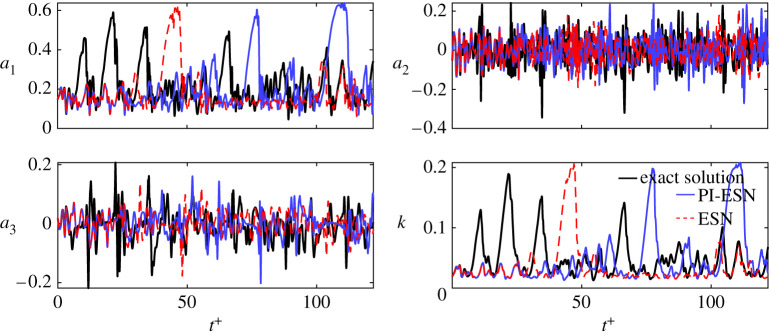
Long-term evolution of the modes’ amplitudes: exact evolution (thick black lines), ESN solution (dashed red lines), PI-ESN solution (blue full lines) with reservoir of 1500 units. (Online version in colour.)

A long-term prediction of the modes’ amplitudes from the trained ESN and PI-ESN is shown in figure 3 for a reservoir of 1500 units. This prediction is made for 10 000 time units, which corresponds to approximately 240 Lyapunov times. Out of that time-series, the first 5000 time units are shown in figure 3. Both the ESN and the PI-ESN qualitatively reproduce the dynamics of the MFE model. In particular, both the ESN and PI-ESN exhibit transition towards a quasi-laminar state with large growth of the first mode, *a*_1_, and an associated increase in the kinetic energy, *k*.

**Figure 4.**
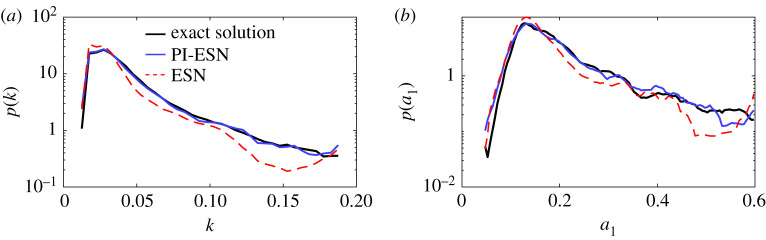
Probability density function of (*a*) turbulent kinetic energy, *k*, and (*b*) first modal coefficient, *a*_1_, of the long-term evolution: exact evolution (thick black line), echo state network (dashed red lines) and physics-informed echo state network (blue full line). (Online version in colour.)

A statistical assessment of the performance of the PI-ESN is analysed in terms of the probability density function (PDF) of *k* and *a*_1_ (figure 4). The PDFs are computed from a time series of 240 Lyapunov times, which is longer than the time series of figure 3 to ensure statistical convergence. The PI-ESN captures more accurately the tail of the PDFs of *k* and *a*_1_ as compared to the data-only ESN. This means that constraining the physics reproduces more accurately the occurrence of extreme events (associated with large values of *a*_1_ and *k*) from a statistical point of view.

**Figure 5.**
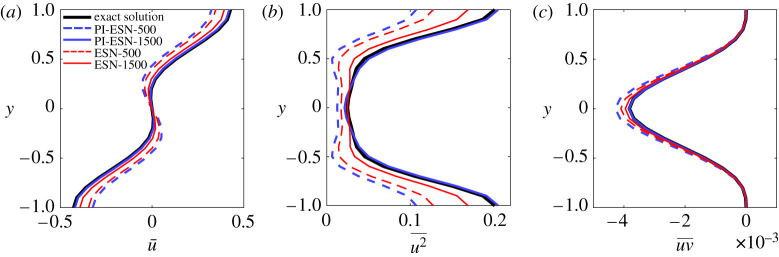
Profile of (*a*) mean streamwise velocity, (*b*) variance and (*c*) mean Reynolds stress. (Online version in colour.)

An additional quantitative comparison, with the statistics of the velocity field, is shown in figure 5 for ESNs and PI-ESNs of different reservoir sizes. The statistics, which are collected for 5000 time units, are computed by averaging the velocity along the two periodic directions, *x* and *z*, and time.

Figure 5 shows that, for small reservoirs (500 units), both the ESN and PI-ESNs do not accurately reproduce the mean streamwise velocity profile and the variance. This is because there are not enough neurons in the reservoir to correctly reproduce the intricate dynamics of the MFE model and its long-term evolution. However, as the reservoir size increases to 1500 units, the PI-ESN accurately captures the mean velocity profile, the variance and the Reynolds stress. Larger reservoirs, with up to 3000 units, have an accuracy that is similar to the reservoir with 1500 units. Therefore, the resulting velocity profiles are shown only for 1500 units in figure 5. On the other hand, the data-only ESN solution is less accurate. A larger reservoir of 2500 units is required for the data-only ESN to accurately reproduce the statistics of the MFE model (result not shown). This highlights how the physical constraint augments the information used during the training of the PI-ESN allowing it to better capture the long-term evolution compared to a data-only ESN. The PI-ESN approach needs a relatively small training dataset of 2500 time units to learn the long-term statistics of the system. Compared to previous studies with LSTM units, these training data are two to three orders of magnitude smaller [15].

### (b) Short-term extreme events prediction

**Figure 6.**
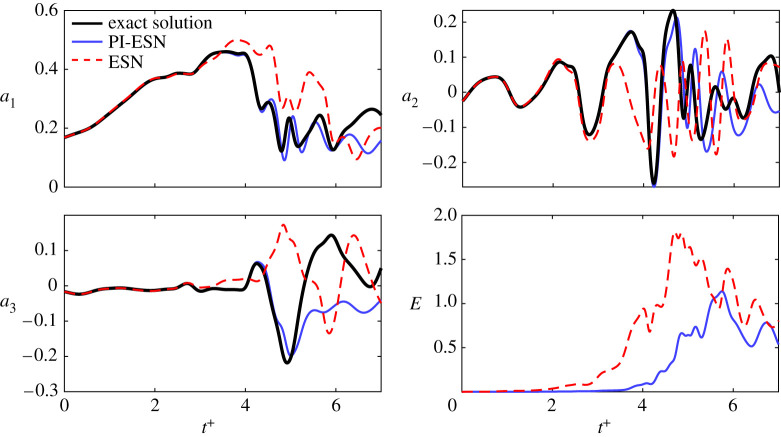
Evolution of modes *a*_1_, *a*_2_, *a*_3_ during the extreme event of figure 1: exact evolution in solid black line; prediction with PI-ESN in solid blue line; and prediction with fully data-driven ESN in dashed red line. The reservoir has *N*_*x*_ = 3000 neurons. *E* is the error (equation (4.1)). (Online version in colour.)

Figure 6 shows the evolution of three modes during the extreme event in the dashed red box of the top panel of figure 1. The PI-ESN solution (solid blue line) and the conventional ESN solution (dashed red line) are computed with a reservoir of *N*_*x*_ = 3000 units. Both solutions are compared against the exact solution from numerical integration (solid black line). The normalized error between the exact evolution and the PI-ESN/ESN predictions is computed as

**Figure 7.**
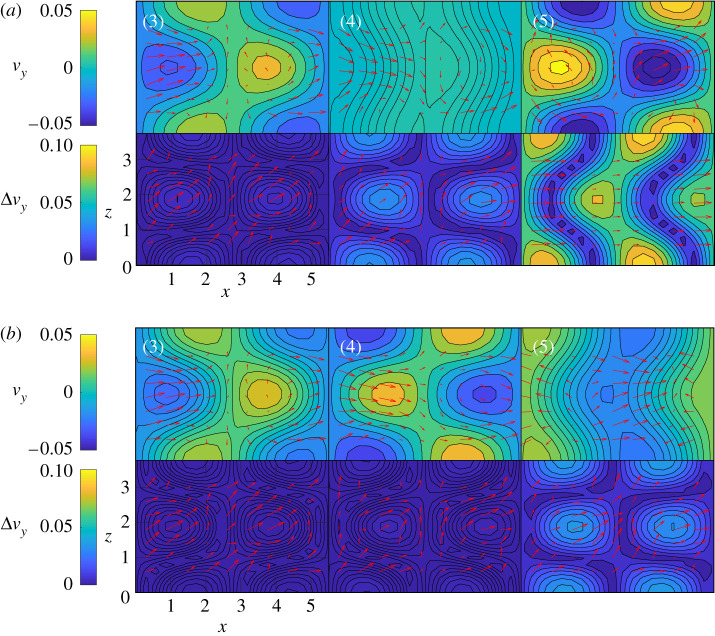
Evolution of the velocity field (top rows) and the normalized error on the out-of-plane velocity (bottom rows) in the velocity field in the mid-*y* plane at the same time instants as panels (3)–(5) of figure 1. Predictions from (*a*) the conventional ESN and (*b*) the PI-ESN. The arrows indicate the in-plane velocity (*x*–*z* directions) and the coloured contour indicates the out-of-plane velocity. The panels correspond to *t*^+^ ≈ [2.14, 3.21, 4.27] of figure 6. (Online version in colour.)

4.1 $$E(n)=\frac{||y(n)-\hat{y}(n)||}{\sqrt{\frac{1}{N_{t}}\sum_{n=1}^{N_{t}}||y(n)||}},$$

where the denominator of the error cannot be zero because the fixed point of the MFE model has ||***y***|| = 1 and the system has unsteady chaotic oscillations. Although the same training data are used for both the PI-ESN and the conventional ESN, the PI-ESN has a significantly higher capability in accurately predicting autonomously the real evolution than the conventional ESN. To compare the performance, we define the predictability horizon as the time required for *E* to exceed the threshold 0.2 from the same initial condition. The predictability horizon of the PI-ESN is ∼2 Lyapunov times longer than the predictability horizon of the conventional ESN. This improvement is achieved by enforcing the prior physical knowledge of the flow, whose evolution must fulfil the momentum and mass conservation laws. As shown in figure 6, until *t*^+^ ≈ 2.14, both ESN and PI-ESN accurately predict the flow evolution. The predicted solution from the conventional ESN starts diverging from the exact evolution at *t*^+^ ≈ 3.21, which leads to a completely different solution during the extreme event. On the other hand, the PI-ESN is able to time-accurately predict the occurrence and the amplitude of the extreme event. After the event has occurred, the solution diverges because the butterfly effect is significant. The velocity fields predicted by the conventional ESN and PI-ESN are shown in figure 7*a*,*b*, respectively. The solutions are shown at the same times as the exact solution in panels (3–5) of figure 1. The bottom rows of figure 7*a*,*b* show the normalized absolute error between the predicted velocity field and the exact velocity field. The discrepancy in the velocity field is mainly due to the error on the prediction of the downstream vortex mode, *a*_3_ (figure 6). On the one hand, because no physical knowledge is constrained in the conventional ESN, the sign and amplitude of *a*_3_ are incorrectly predicted, which means that the out-of-plane velocity evolves in the opposite direction of the exact solution. On the other hand, the PI-ESN is able to predict satisfactorily both the in-plane velocity and the out-of-plane velocity during the extreme event.

**Figure 8.**
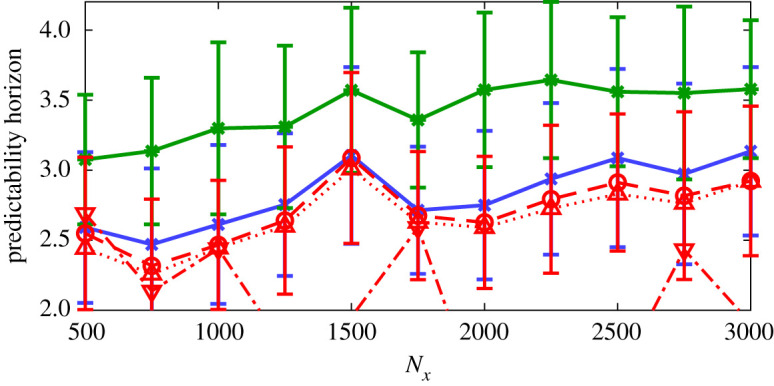
Comparison of the average predictability horizons of extreme events from the PI-ESN with original hyperparameters (full blue line with crosses), the PI-ESN with optimized hyperparameters (full green line with stars), ESN with *γ* = 10^−6^ (dashed red line with circles), *γ* = 10^−5^ (dotted red line with upward triangles) and *γ* = 10^−7^ (dash-dotted line with downward triangles) for the prediction of 50 extreme events. The error bars show half of the standard deviation. *N*_*x*_ is the number of neurons. (Online version in colour.)

To quantitatively assess the robustness of these results, we compute the average predictability horizon of the machines with no further training. We follow the following steps: (i) by inspection of our dataset (a subset of which is shown in figure 1), we define events as extreme when their kinetic energy is *k* ≥ 0.1; (ii) we identify the times at which all the extreme events start in our dataset (50 extreme events are identified); (iii) for each time, the exact initial condition at *t*^+^ ≈ 0.61 just before the time instant in which the extreme events starts is inputted in the PI-ESN and ESN; (iv) the machines are time evolved to provide the prediction; and (v) the predictability time is computed by averaging over all the extreme events in the dataset. The mean predictability time and the standard deviation are computed with validation data containing 50 extreme events. The results are parametrized with the size of the reservoir, *N*_*x*_ (figure 8). On the one hand, for small reservoirs ($N_{x}≲2000$), the performances of the ESN and PI-ESN are comparable. This means that the performance is more sensitive to the data cost functional, *E*_*d*_, than the physical error, *E*_*p*_. On the other hand, for larger reservoirs ($N_{x}≳2000$), the physical knowledge is fully exploited by the PI-ESN. This means that the performance becomes markedly sensitive to the physical error, *E*_*p*_. This results in an improvement in the average predictability of up to ≈1.5 Lyapunov times. Because an extreme event takes ≈3 Lyapunov time on average, the improved predictability time of the PI-ESN is key to the time-accurate prediction of the occurrence and amplitude of the extreme events. To further assess the accuracy of the PI-ESN with respect to the data-only ESN, we analyse data-only ESNs trained with different Tikhonov regularization factors (*γ* = 10^−5^ and *γ* = 10^−7^) in figure 8. The PI-ESN outperforms the data-only ESN.

For completeness, we re-tune the hyperparameter of the PI-ESN by performing a grid search, which provides the optimal parameters Λ=0.7, *σ*_in_ = 0.9, 〈*d*〉 = 3. The extreme event prediction horizon for the PI-ESN with these new optimal hyperparameters is shown in figure 8 (in green). A higher prediction horizon can be achieved with the hyperparameter re-tuning.

### (c) Robustness to noisy data

Because the accuracy of the ESN and PI-ESN—and more generally, of any data-driven method—depends on the quality of the training data, the effect of additive noise is analysed. Noise is added to the training dataset presented in figure 1 with signal-to-noise ratios (SNR) of 30 dB and 40 dB, which are representative of noise in experiments [43]. The ESN and PI-ESN are trained on the noisy data, and the accuracy of the long- and short-term prediction is analysed. The dataset length and hyperparameters are the same as those of §§4a and 4b. Here, because the training dataset is small (2500 time units, which correspond to 60 Lyapunov times), noise has a marked (detrimental) impact on the training of the ESN for learning the physical dynamics. (By contrast, when the training dataset is large—i.e. the data contain sufficient physical dynamics for the machine to be trained—a small addition of noise can have a positive effect on the robustness of the ESN [41].)

**Figure 9.**
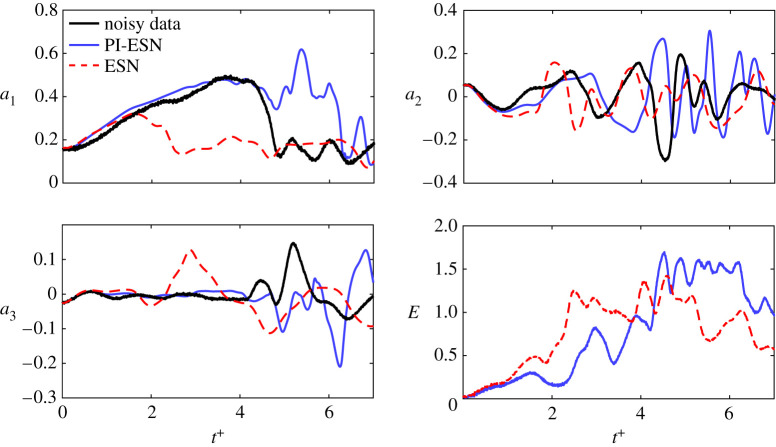
Evolution of *a*_1_, *a*_2_, *a*_3_ during the extreme event of figure 1: noisy evolution in solid black line; prediction with PI-ESN in solid blue line; and prediction with fully data-driven ESN in dashed red line. The reservoir has *N*_*x*_ = 3000 neurons. *E* is the error (equation (4.1)). The noisy data correspond to the case with SNR = 30 dB. (Online version in colour.)

Figure 9 shows the evolution of three modes, the normalized error and the noisy solution. Similarly to the noise-free case (figure 6), the PI-ESN is quantitatively more predictive than the data-only ESN. For example, the PI-ESN predicts the growth of the *a*_1_ mode, which is physically the quasi-laminar profile forming during the extreme event. Although after 4 Lyapunov times the PI-ESN diverges due to the high sensitivity of chaotic flows to noise, the overall statistics are well predicted, as discussed next. One possible strategy to improve the performance is to increase the number of collocation points in the training of the PI-ESN.

**Figure 10.**
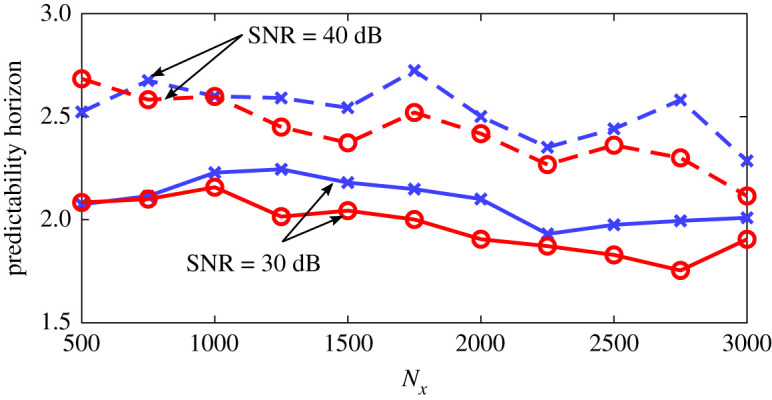
Comparison of the average predictability horizons of extreme events from the PI-ESN (blue full and dashed lines with crosses) and ESN (red full and dashed lines with circles) for all the extreme events in the dataset when trained with noisy database. Full lines: SNR = 40 dB, dashed lines: SNR = 30 dB. *N*_*x*_ is the number of neurons. (Online version in colour.)

The statistical assessment is shown in figure 10. Compared to the noise-free case of §4b, the predictability time of extreme events becomes smaller as the noise level becomes larger. This loss in accuracy is due to the noise in the training data. Both the ESN and PI-ESN minimize the error on the noisy data, *E*_*d*_, which causes the machines to try to fit the noisy data, resulting in the reduction of predictive capability. This loss becomes more significant as the noise increases. If the number of neurons in the reservoir is further increased, the ESN and, to a lesser extent, the PI-ESN will start overfitting the noise. The PI-ESN mitigates the loss of accuracy through the physical loss, *E*_*p*_, which acts as a regularization term that filters out the noise. This allows the PI-ESN to maintain a higher accuracy in the prediction of the extreme event (figure 6). As a result, the PI-ESN provides a longer accurate prediction of the extreme events, by up to 0.4 Lyapunov time, compared to the data-only ESN. This effect becomes more significant as the number of neurons in the reservoir increases. In other words, the physical constraint in the PI-ESN acts as a denoizer. In the presence of noise, the PI-ESN does not attempt to fit the noisy data, but it balances the fit with the physics. The physical loss allows the PI-ESN to determine an appropriate physical prediction, which deviates from the noisy data to approach the physical dynamics. This denoizing property of the PI-ESN is consistent with previous studies [44,45]. This overcomes the lack of robustness of the data-only ESN, which cannot discriminate the noise from the actual flow dynamics.

**Figure 11.**
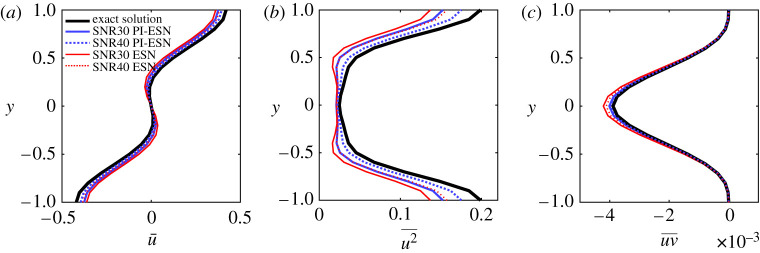
Profile of (*a*) mean streamwise velocity, (*b*) variance and (*c*) mean Reynolds stress. (Online version in colour.)

Finally, figure 11 compares the statistics of the velocity obtained from a long-term prediction of 5000 time units for a reservoir size of 1500 units (similarly to the noise-free case of figure 5). Consistently with the short-term performance, the accuracy on the statistics decreases as the noise level increases in the training data. For both noise levels, the PI-ESN outperforms the data-only ESN.

## 5. Final discussion and future directions

We propose a PI-ESN, which combines empirical modelling, based on reservoir computing, with physical modelling, based on conservation laws, to time-accurately predict extreme events (short-term dynamics) and the statistics (long-term dynamics) of a chaotic flow. We compare the performance of the PI-ESN with a fully-data driven ESN. The former is a physics-constrained machine, whereas the latter is a physics-unaware machine because it is trained with data only.

In the PI-ESN, the physical error from the conservation laws is minimized beyond the training data. This brings in crucial information, which is exploited in two ways: (i) with the same amount of available data, the PI-ESN solution is accurate for a longer time than the conventional ESN solution, i.e. the information from physical knowledge enhances the accuracy of the autonomous prediction of the PI-ESN, and (ii) fewer data are required to obtain the same accuracy as the conventional ESN. Here, we take advantage of property (i) for the prediction of extreme events in a model of turbulence, and the statistics of velocity. Extreme events may be generally rare, which makes it difficult for physics-unaware data-driven methods to be trained. By contrast, constraining the physics enables the PI-ESN to predict chaotic dynamics that cannot be inferred from data only. Additionally, the PI-ESN well captures the long-term flow statistics with relatively small training data. Finally, the approach also shows robustness with respect to noise.

It should be noted that the framework proposed in this paper relies on knowing the governing equations of the system studied, which is an ideal case. Cases when this knowledge is not perfect, for example, when only approximate equations are available, will constitute the scope for future work. In addition, the possibility of applying the proposed method to larger dimensional flow systems, such as the full Navier–Stokes equations, will also be explored. This can be achieved by using autoencoders to learn the spatial correlations, as shown in [46]. These avenues will be investigated in future research.

## Data accessibility

The data related to this publication are available at https://gitlab.tudelft.nl/adoan/pi-esn.
